# Temporal phylogeography of *Yersinia pestis* in Madagascar: Insights into the long-term maintenance of plague

**DOI:** 10.1371/journal.pntd.0005887

**Published:** 2017-09-05

**Authors:** Amy J. Vogler, Voahangy Andrianaivoarimanana, Sandra Telfer, Carina M. Hall, Jason W. Sahl, Crystal M. Hepp, Heather Centner, Genevieve Andersen, Dawn N. Birdsell, Lila Rahalison, Roxanne Nottingham, Paul Keim, David M. Wagner, Minoarisoa Rajerison

**Affiliations:** 1 The Pathogen and Microbiome Institute, Northern Arizona University, Flagstaff, Arizona, United States of America; 2 Plague Research Unit, Institut Pasteur de Madagascar, Antananarivo, Madagascar; 3 School of Biological Sciences, University of Aberdeen, Aberdeen, United Kingdom; 4 Pathogen Genomics Division, Translational Genomics Research Institute, Flagstaff, Arizona, United States of America; Beijing Institute of Microbiology and Epidemiology, CHINA

## Abstract

**Background:**

*Yersinia pestis* appears to be maintained in multiple, geographically separate, and phylogenetically distinct subpopulations within the highlands of Madagascar. However, the dynamics of these locally differentiated subpopulations through time are mostly unknown. To address that gap and further inform our understanding of plague epidemiology, we investigated the phylogeography of *Y*. *pestis* in Madagascar over an 18 year period.

**Methodology/Principal findings:**

We generated whole genome sequences for 31 strains and discovered new SNPs that we used in conjunction with previously identified SNPs and variable-number tandem repeats (VNTRs) to genotype 773 Malagasy *Y*. *pestis* samples from 1995 to 2012. We mapped the locations where samples were obtained on a fine geographic scale to examine phylogeographic patterns through time. We identified 18 geographically separate and phylogenetically distinct subpopulations that display spatial and temporal stability, persisting in the same locations over a period of almost two decades. We found that geographic areas with higher levels of topographical relief are associated with greater levels of phylogenetic diversity and that sampling frequency can vary considerably among subpopulations and from year to year. We also found evidence of various *Y*. *pestis* dispersal events, including over long distances, but no evidence that any dispersal events resulted in successful establishment of a transferred genotype in a new location during the examined time period.

**Conclusions/Significance:**

Our analysis suggests that persistent endemic cycles of *Y*. *pestis* transmission within local areas are responsible for the long term maintenance of plague in Madagascar, rather than repeated episodes of wide scale epidemic spread. Landscape likely plays a role in maintaining *Y*. *pestis* subpopulations in Madagascar, with increased topographical relief associated with increased levels of localized differentiation. Local ecological factors likely affect the dynamics of individual subpopulations and the associated likelihood of observing human plague cases in a given year in a particular location.

## Author summary

Plague exists in several geographically separate phylogenetic groups within Madagascar, but little is known about the dynamics of these groups through time. We subtyped 773 Malagasy plague samples and identified 18 phylogenetic groups that showed persistence in the same locations over a period of almost two decades. Locations with more topographical relief contained more phylogenetic groups than flatter areas and different phylogenetic groups varied considerably in the number of samples collected each year. Transfers of plague from one location to another definitely occur, but appear to seldom result in the transplanted phylogenetic group becoming successfully established in a new location. Persistent, local transmission cycles are likely responsible for the long term maintenance of plague in Madagascar, rather than repeated wide scale disease transmission events. Landscape likely plays a role in maintaining different phylogenetic groups, with increased topographical relief associated with increased numbers of phylogenetic groups. Local ecological factors likely affect the dynamics of individual subpopulations and the associated likelihood of observing human plague cases in a given year in a particular location.

## Introduction

*Yersinia pestis* is one of the most successful bacterial pathogens known. Its most recent common ancestor (MRCA) may have emerged less than 6,000 years ago yet it appears to have been widely dispersed throughout Eurasia during the Bronze Age. Following the acquisition of some key genetic changes only ~3000 years ago, some *Y*. *pestis* became capable of causing the deadly, flea-borne bubonic plague [1] and then swept the known world in three recognized pandemics [2]. These pandemics likely originated from Asia in multiple successive waves [3, 4], causing hundreds of millions of human deaths and establishing a number of global enzootic foci [2]. Indeed, *Y*. *pestis* has successfully spread to every continent except Antarctica and currently has established enzootic foci in Asia, Africa, and the Americas [2, 5]. *Y*. *pestis* is thought to persist in these enzootic foci through low-level cycling in numerous, often cryptic, rodent species whose populations either include a mixture of relatively resistant and highly susceptible individuals or are characterized by a high replacement rate. Periodically, *Y*. *pestis* emerges from these enzootic reservoirs in large-scale epizootics involving massive die-offs of highly susceptible rodent species that serve to amplify and spread *Y*. *pestis* [2, 6, 7].

Alternating enzootic and epizootic cycles, human involvement, and ecology have all contributed to the observed phylogeography of *Y*. *pestis* and its consistent pattern of the spread of one to a few genotypes followed by localized differentiation. Globally, this pattern is most clearly observed with the highly successful 1.ORI population, which was responsible for the third pandemic. In the global *Y*. *pestis* phylogeny, the basal node of this population is characterized by a large polytomy, suggesting that a rapid expansion preceded the spread of this group around the world [3]. Numerous independent lineages branch off from this initial polytomy, reflecting the independent evolution of *Y*. *pestis* in the many new enzootic foci that were established after this event [3, 4]. Importantly, the global spread of this group would not have been possible without the inadvertent human transport, via steamship and other means, of rats and fleas infected with *Y*. *pestis* [2, 5]. In addition, the local ecology of regions where *Y*. *pestis* was introduced strongly influenced the establishment of stable enzootic foci. During the third pandemic, new plague foci became established in locations that contained either suitable native rodent species (e.g., North America) or a large enough population of non-native rodents (e.g., Madagascar) that could sustain the rodent/flea transmission cycle. In contrast, locations without these conditions (e.g., Australia), did not develop enzootic foci, although they did experience outbreaks, which subsided after the number of non-native rodents was reduced, resulting in an evolutionary dead end for *Y*. *pestis* in these locations [5].

The influence of enzootic/epizootic cycling, human involvement, and ecology on *Y*. *pestis* phylogeography is also apparent on a regional level, such as in the well-studied plague foci of Madagascar. Two large areas in the central and northern highlands serve as traditional plague foci in Madagascar [8, 9], with the persistence of *Y*. *pestis* in these areas linked to the presence of two flea vectors, *Xenopsylla cheopis* and *Synopsyllus fonquerniei*, which are less abundant and absent, respectively, at lower elevations [8, 10, 11]. A third focus in the port city of Mahajanga experienced several outbreaks when *Y*. *pestis* was first introduced to Madagascar [8, 12] and then again in the 1990s [8, 13–16]. However, this focus does not appear to be stable, as evidenced by the 62 year gap in observed plague activity between the initial and 1990s outbreaks [8, 13–16] and the apparent lack of current activity, based on the absence of additional confirmed human cases [8]. Within the traditional foci, *Y*. *pestis* appears to be maintained in multiple, geographically separate, and phylogenetically distinct subpopulations that are likely sustained by the black rat (*Rattus rattus*) [17–19], the primary plague host in rural Madagascar [8–12]. There is also evidence of multiple, likely human-mediated, long-distance dispersal events of different genotypes to new locations, with at least one such event responsible for the re-emergence of the Mahajanga focus during the 1990s [18, 19].

The spread of one to a few genotypes followed by localized differentiation is a well-established phylogeographic pattern of *Y*. *pestis*, at multiple geographic scales [3, 4, 18–20]. In Madagascar, there are multiple, geographically and phylogenetically distinct subpopulations that have arisen due to this localized differentiation [17–19]. However, the dynamics of these locally differentiated subpopulations through time are mostly unknown. Previous studies have suggested that some subpopulations experience extinction and/or decreases in frequency and that new subpopulations emerge and spread, potentially becoming established in new locations, either temporarily or more long-term [17–19]. In addition, a temporal study of the 1990s Mahajanga outbreaks depicted a striking cycling pattern of diversity generation and loss that occurred during and after each outbreak, consistent with severe inter-seasonal genetic bottlenecks and large seasonal population expansions [18]. However, this type of concerted temporal analysis has not been attempted in the traditional foci.

Here, we investigate the phylogeography of *Y*. *pestis* in Madagascar over an 18 year period from 1995 to 2012. We generated whole genome sequences for an additional 31 strains, enabling us to use a total of 37 Malagasy strain sequences to discover additional SNPs that we used in conjunction with previously identified SNPs and multiple-locus variable-number tandem repeat (VNTR) analysis (MLVA) to genotype 773 Malagasy *Y*. *pestis* samples from 1995 to 2012. We then spatially map these samples through time on a fine geographic scale to examine *Y*. *pestis* phylogeographic patterns in Madagascar through time.

## Methods

### Ethics statement

The DNAs used in this study (S1 Table) were extracted from *Y*. *pestis* cultures or complex human clinical samples originally isolated or collected, respectively, by the Malagasy Central Laboratory for Plague and Institut Pasteur de Madagascar as part of Madagascar’s national plague surveillance plan overseen by the Malagasy Ministry of Health. This program requires declaration of all suspected human plague cases and collection of biological samples from those cases. These samples and any cultures or DNA derived from those samples are all de-linked from the patients from whom they originated and analyzed anonymously if used in any research study, such as this one. The Northern Arizona University Institutional Review Board did not require additional review of this research due to the anonymous nature of the samples.

### DNAs

DNA was obtained from 773 *Y*. *pestis* strains or complex human clinical samples collected from 1995 through 2012 (S1 Table). Geographical origin data for these samples was very comprehensive, including at least commune and district of origin, with most (N = 729) also including the fokontany (i.e., village) of origin (each commune is divided into fokontany). The samples originated from 384 fokontany, from 175 communes, in 32 districts in Madagascar (See S1 Fig for a map of the sampled districts). The DNAs included 173 and 85 samples that were previously analyzed in references [18, 19] and [17], respectively (S1 Table). The remaining 515 novel DNAs were extracted from strains selected to emphasize districts Betafo, Mandoto, Antsirabe I, Antsirabe II, and some neighboring areas (hereafter referred to as the Betafo region), which experience some of the highest human plague case incidence rates in Madagascar; and district Moramanga and neighboring areas (hereafter referred to as the Moramanga region), which also declares human plague cases nearly every year, but at a lower frequency than the Betafo region (S1 Fig). Indeed, the analysis of these areas was very comprehensive, including all of the available samples from the Betafo, Mandoto, Antsirabe I, Antsirabe II, and Moramanga districts, and subsets of samples from the surrounding districts over the 18 year study period (S1 Fig, S1 Table). DNAs consisted of simple heat lysis preparations, extracts prepared using the QIAamp DNA Mini Kit (Qiagen, Hilden, Germany), or whole genome amplification (WGA, QIAGEN, Valencia, CA) products generated from the heat lysis or kit extraction preps. Most of the samples (90%) were obtained from human plague cases with a smaller number collected from other mammals or fleas (S1 Table).

### Existing SNP and MLVA screening

All DNAs were genotyped, as previously described, using 63 assorted, previously identified SNPs [18, 19] and a 43-locus MLVA [20]. Screened SNPs included Mad-08 through Mad-48 from reference [19] and Mad-57 through Mad-78 from reference [18] (S2 Table). These SNPs were screened in a hierarchical fashion, with SNP Mad-43 screened first to determine if a sample belonged in Group I or II, two previously described major groups in Madagascar [4, 19], and then additional, appropriate Group I or II SNPs screened to determine which previously described SNP-defined group (i.e., node) a sample belonged to. MLVA was then used to provide additional discrimination within each node.

### Whole genome sequencing and SNP discovery

A total of 31 strains were selected for whole genome sequencing to identify additional SNPs for phylogenetic analysis (S1 Table). These strains were chosen based on the quality of the available DNA (e.g., DNAs extracted from complex human clinical samples and many of the heat lysis preps proved unsuitable for whole genome sequencing due to low concentrations) and also to maximize the potential for new node discovery by selecting phylogenetically diverse strains with an emphasis on existing nodes containing larger numbers of samples, as determined from the above SNP and MLVA analyses (S2 Fig) [21]. Illumina sequence libraries were prepared as previously described [22] and the new genomes sequenced on the Illumina HiSeq platform (Illumina, San Diego, CA), producing 2 × 100 bp reads. Paired-end Illumina whole genome sequence data for each newly sequenced strain and previously published whole genome sequences for six other Malagasy strains (MG05-1020 [GenBank: AAYS00000000], IP275 [GenBank: AAOS00000000], 53/91, 64/91, 154/98 B, 17/99 B [GenBank: SRP017903]) [4, 18] were aligned using BWA-MEM v0.7.5 against the published genome for strain CO92 [23–25]. Duplicate regions were identified and removed based on a self-alignment of the CO92 genome using NUCmer v3.23 [26]. SNPs were called on the binary alignment map (BAM) file [27] using the UnifiedGenotyper method in GATK v2.7.5 [28, 29]. SNPs below a minimum depth (10x) or minimum allele proportion (90%) were removed from subsequent analyses. Alignment and SNP calling methods were wrapped by the Northern Arizona SNP Pipeline (NASP) (http://tgennorth.github.io/NASP/) [30].

### New SNP screening

Primers were designed targeting a ~250 bp region around each of 188 newly identified potential SNP targets (S3 Table) using Primer3 [31, 32] with strain CO92 as the reference sequence [24] and with the potential SNP located at the center of each amplicon. Primer sets were ordered from IDT (Coralville, IA) and contained universal tails used to anneal unique indexes for sample barcoding (forward primers, UT1 = 5’-ACCCAACTGAATGGAGC-3’and reverse primers, UT2 = 5’-ACGCACTTGACTTGTCTTC-3’) [33]. Assays were grouped into one of four multiplex PCRs, with 29 assays in mix 1, 63 assays in mix 2, 65 assays in mix 3, and 31 assays in mix 4 (S3 Table). Each multiplex was validated in singleplex using SYBR real-time PCR with the multiplex PCR used as template [34]. The optimized multiplexes were then screened across 864 total samples (773 of which were analyzed here) (S1 Table). A single 10 μL multiplex PCR reaction consisted of final concentrations of the following reagents: 1x 10x PCR buffer, 1.5 mM MgCl_2_, 0.2 mM dNTPs, 0.4 μM of each primer, 1.5 units of Platinum Taq (Invitrogen, Grand Island, NY), and 1 μL of template. Multiplex PCR cycle conditions consisted of 95°C, 10 min; (94°C, 30 sec; 55°C, 30 sec; 72°C, 30 sec) × 40 cycles; 72°C, 5 min; held at 10°C.

Amplicon libraries were prepared using universal tails as previously described [33]. Briefly, a cleanup was performed on the multiplex PCR products using a 1:1 bead ratio to PCR product of 1x Agencourt AMPure XP beads (Beckman Coulter, Indianapolis, IN) with elution in 30 μL of a 10 mM Tris-HCl 0.05% Tween 20 solution. Indexed barcodes were then applied to each sample, providing a unique barcode to identify each sample. The Index Extension PCR was a single 25 μL PCR containing 12.5 μL of 2x KAPA HiFi HotStart ReadyMix (Kapa Biosystems, Wilmington, MA), 1 μL 10 μM common universal tail primer, 1 μL 10 μM specific index universal tail primer, 8.5 μL molecular grade water, and 2 μL cleaned up PCR product. Extension PCR parameters consisted of 98°C, 2 min; (98°C, 30 sec; 60°C, 20 sec; 72°C, 30 sec) × 6 cycles; 72°C, 5 min; held at 10°C. Following index addition, the PCR product was cleaned up again using a 1:1 bead ratio with Agencourt AMPure XP beads using an elution of 40 μL of a 10 mM Tris-HCl 0.05% Tween 20 solution.

Amplicon libraries were normalized to a concentration of 25 nM using the SequalPrep Normalization Plate Kit, 96-well (Thermo Fisher Scientific, Waltham, MA) according to manufacturer’s instructions. Following normalization, the amplicon libraries were pooled in sets of 96 samples using 5 μL from each of the multiplexes, resulting in nine pools of 96 uniquely barcoded samples across 188 targets. Final sample pools were generated by pooling 100 μL from each of three of the nine plate pools into a single tube, for three final pools of 288 samples each. The three final pools were concentrated by conducting another cleanup using a 1:1 bead ratio with Agencourt AMPure XP beads and an elution of 30 μL of a 10 mM Tris-HCl 0.05% Tween 20 solution. These final, concentrated pools were then sequenced on the Illumina MiSeq platform using 2 × 300 bp version 3 sequencing chemistry (Illumina, San Diego, CA).

Amplicon sequences were aligned to the reference genome of strain CO92 [24] using BWA-MEM [23] and SNPs were called with the UnifiedGenotyper method in GATK [28, 29] in conjunction with the NASP pipeline (http://tgennorth.github.io/NASP) [30]. The resulting SNP matrix was filtered to focus on the SNPs to be verified.

### Phylogeographic analyses

**Fig 1:**
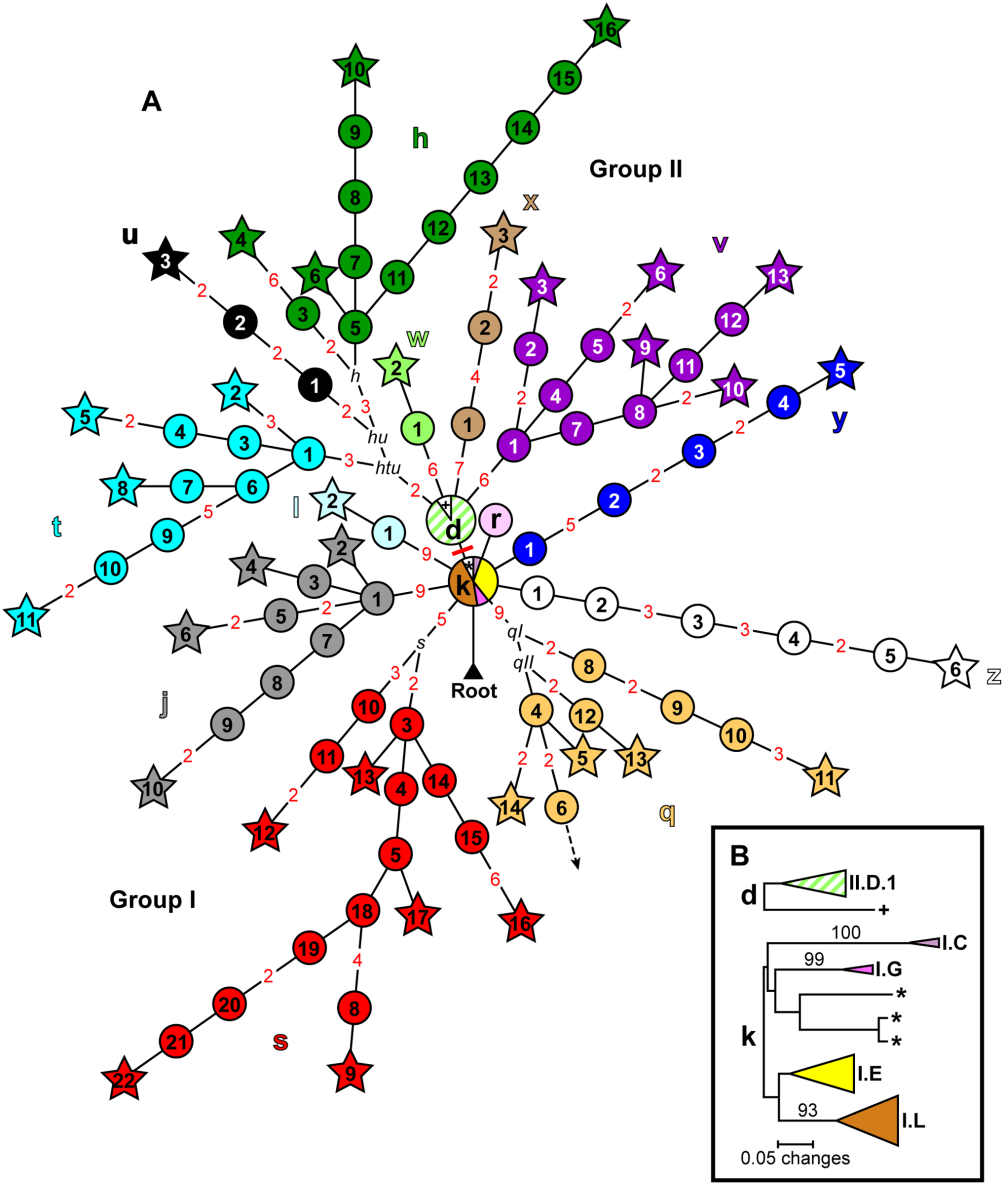
SNP and MLVA phylogenies depicting 18 major phylogenetic subgroups identified among 773 Malagasy *Yersinia pestis* samples. (A) SNP phylogeny based on 212 informative SNPs identifying 100 individual nodes (circles and stars) among 770 Malagasy *Y*. *pestis* samples (only the lineage could be identified for the remaining 3 samples, and not the specific node). Stars indicate terminal nodes defined by a sequenced strain. Circles indicate intermediary nodes (i.e., collapsed branch points) along the lineages containing groups of samples. Branch points that did not contain any samples are labeled in black italics. A dashed arrow indicates a branch leading to a single, previously identified, terminal node not represented among the samples in this analysis. Lineages (lower case letters) and nodes within lineages (numbers within circles and stars) were named as in [17–19], with new letters and numbers assigned to newly identified lineages and nodes, respectively. Basal nodes d and k are represented by pie charts, indicating the presence of multiple MLVA identified subgroups within these nodes. Color shading indicates the 18 identified phylogenetic subgroups and, to the extent possible, corresponds to the subgroup colors used in reference [19]. Solid pale green and striped pale green, respectively, indicate the new SNP lineage (w) and remaining subset of samples within basal node d that were split in this analysis from a single previously identified subgroup. The number of SNPs on branches with >1 SNP are indicated in red. The single SNP differentiating between Groups I and II is indicated by a perpendicular red line on the branch between nodes d and k. (B) MLVA phylogenies of 10 and 38 Malagasy *Y*. *pestis* samples from basal SNP nodes d and k, respectively. The MLVA phylogenies consist of neighbor-joining dendrograms constructed in MEGA6 [35] using mean character based distance matrices. Bootstrap values ≥50 (generated in PAUP 4.0b10 (D. Swofford, Sinauer Associates, Inc., Sunderland, MA) based upon 1,000 simulations) supporting MLVA phylogeny branches are indicated. One and four additional phylogenetic subgroups consistent with previous analyses [19] were identified within the MLVA phylogenies of nodes d and k, respectively. In addition, 1 and 3 samples within nodes d and k, respectively, did not fall into any identified phylogenetic subgroup and were labeled with a “+” or an “*”, and classified as II.NONE and I.NONE, respectively.

**Fig 2:**
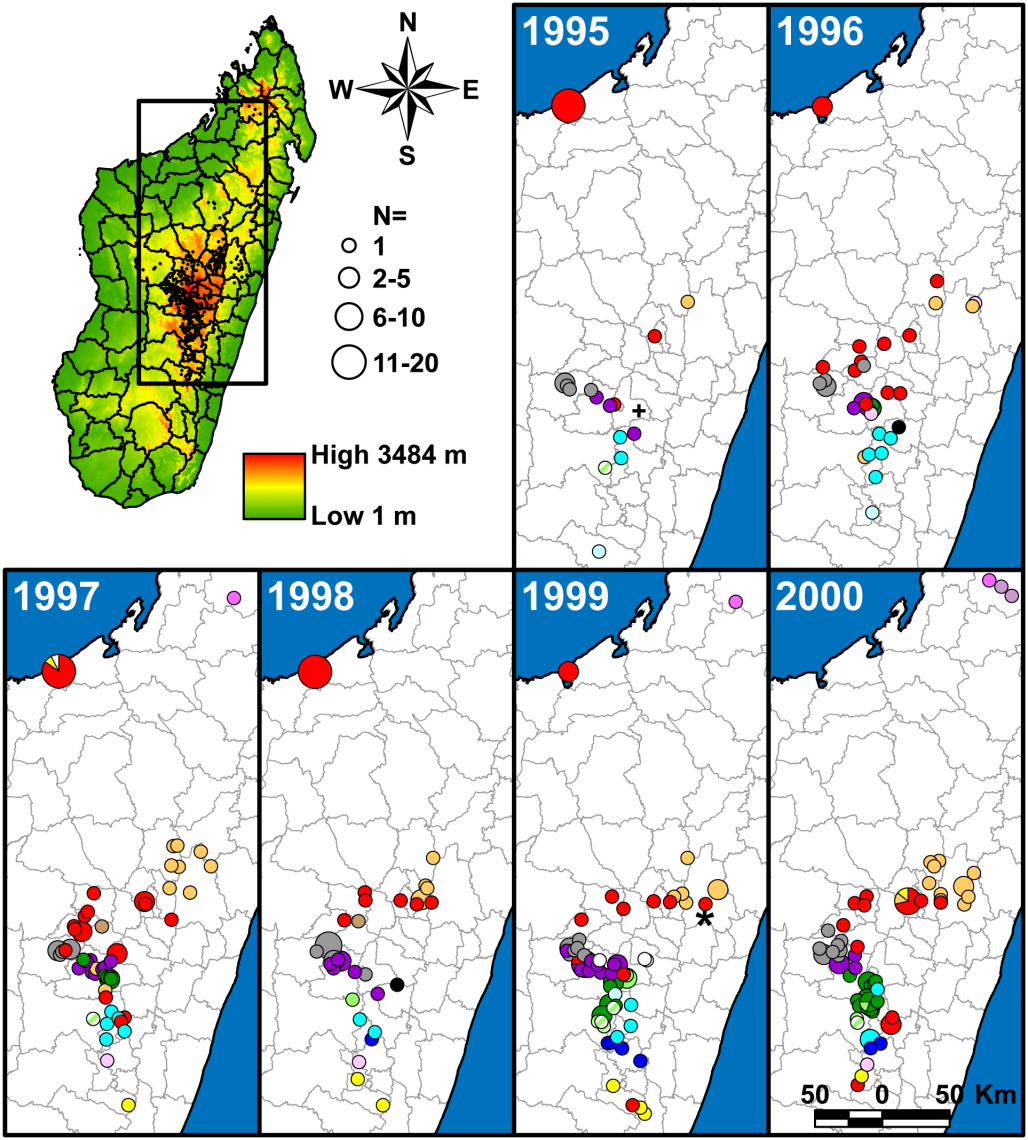
Geographic distribution of 355 Malagasy *Yersinia pestis* samples from 1995 to 2000. The map of Madagascar in the upper left indicates elevation, all of the geographic points in this study (small black points), and the portion of Madagascar represented in the other panels (rectangle). The geographic distribution of identified subgroups is presented in separate panels for each year. Circles in the panels represent the locations of the fokontany (i.e., villages) or commune centroids (when the fokontany was unknown) where samples were collected. In some cases where separate circles were too close together to be visibly distinguished at this scale, a single circle indicating the overlapping circles was substituted. This occurred primarily for fokontany within communes Mahajanga and Mahabibo within district Mahajanga I, and for fokontany within the various arrondissements (i.e., administrative divisions) within district Antananarivo Renivohitra, but also occasionally occurred at other locations. Colors of the mapped circles indicate identified subgroups and correspond to the subgroup color designations in Fig 1. Divisions within circles indicate that multiple subgroups were found at that location in that year. Unaffiliated Group I and II samples (i.e., I.NONE and II.NONE) are indicated by a “*” and a “+”, respectively.

**Fig 3:**
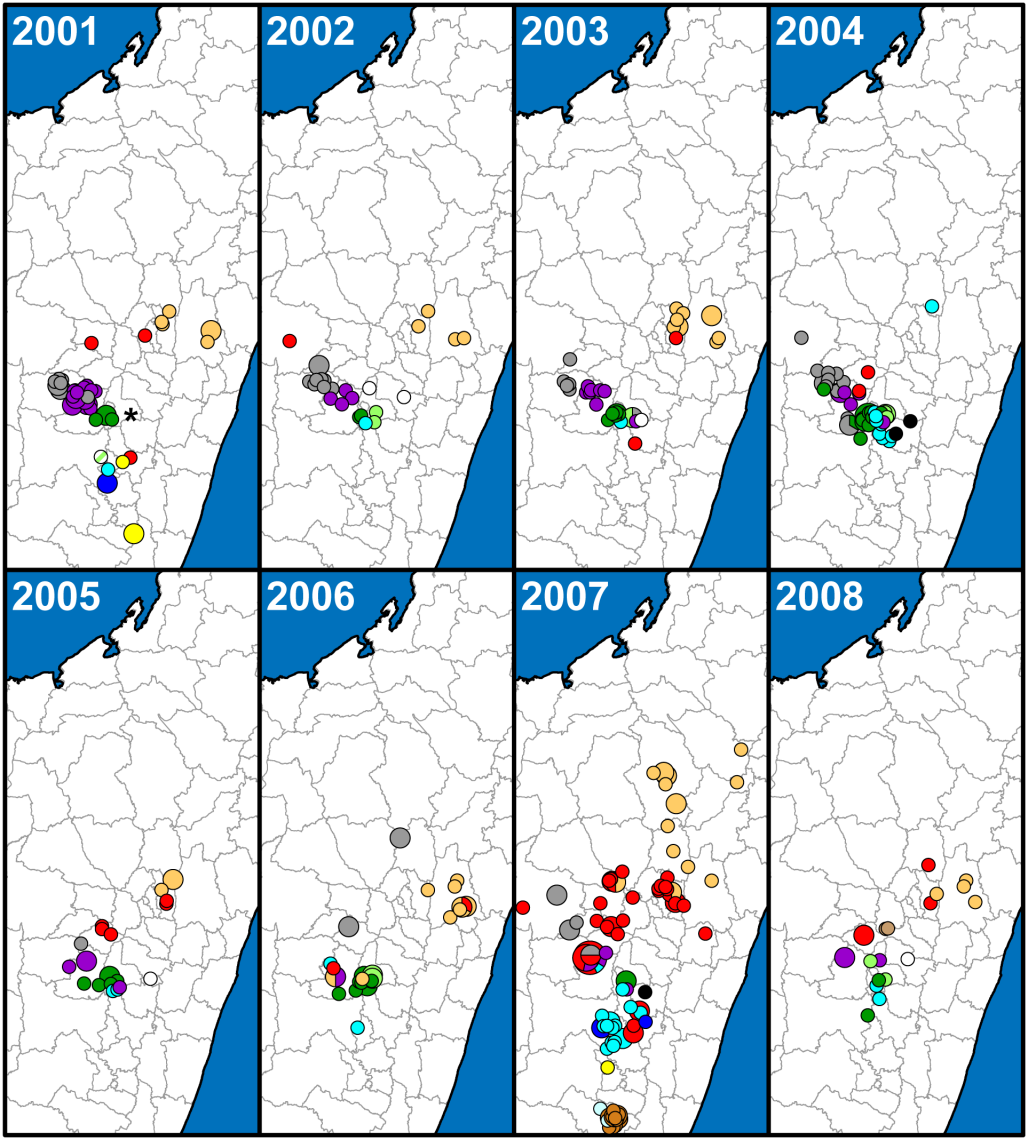
Geographic distribution of 373 Malagasy *Yersinia pestis* samples from 2001 to 2008. The geographic distribution of identified subgroups is presented in separate panels for each year, with symbols and colors as in Fig 2.

**Fig 4:**
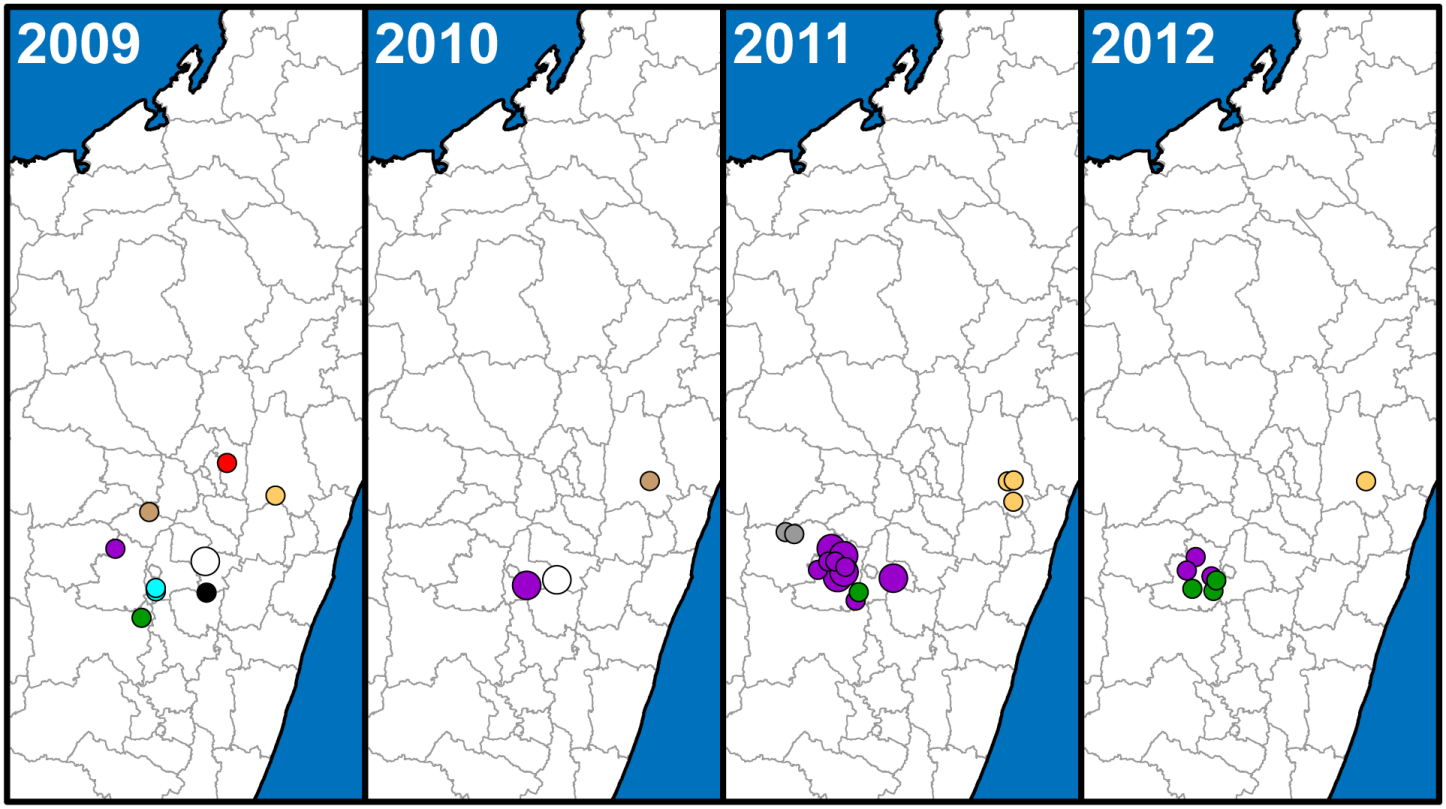
Geographic distribution of 45 Malagasy *Yersinia pestis* samples from 2009 to 2012. The geographic distribution of identified subgroups is presented in separate panels for each year, with symbols and colors as in Fig 2.

**Fig 5:**
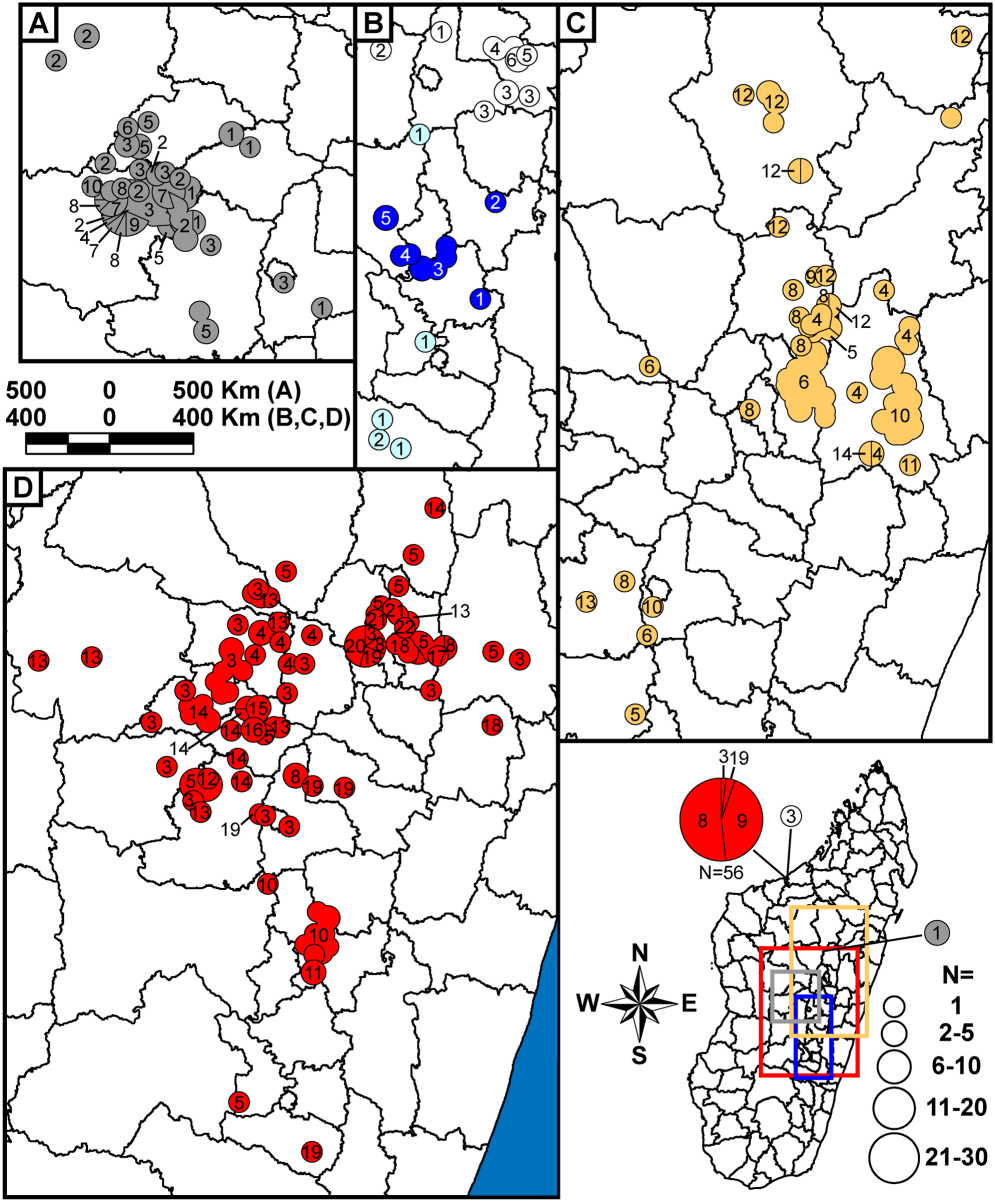
Geographic distributions of identified SNP nodes within Group I. Four map panels showing the geographic distributions of identified SNP nodes within Group I phylogenetic subgroups j (A); l, y, and z (B); q (C); and s (D) are presented. A full map of Madagascar in the lower right indicates the portion of Madagascar included in each of the expanded map panels, with each expanded section indicated by a colored rectangle corresponding to the color associated with one of the phylogenetic subgroups depicted in the corresponding expanded panel. Circles within the expanded map panels represent the locations of the fokontanys (i.e., villages) or commune centroids (when the fokontany was unknown) where samples were collected. In some cases where separate circles were too close together to be visibly distinguished at this scale, a single circle indicating the overlapping circles was substituted. This occurred primarily for fokontany within communes Mahajanga and Mahabibo within district Mahajanga I, and for fokontany within the various arrondissements (i.e., administrative divisions) within district Antananarivo Renivohitra, but also occasionally occurred at other locations. Colors within the mapped circles correspond to the subgroup color designations in Fig 1. Divisions within circles indicate that multiple SNP determined nodes were found at that location. Numbers within circles and pie chart slices indicate the node within a given subgroup from Fig 1A that the mapped samples belong to. Overlapping circles and pie chart slices representing the same node were merged together and identified with a single label to simplify the maps. The specific node could not be definitively determined for two subgroup q samples and one subgroup s sample, so no number is shown.

**Fig 6:**
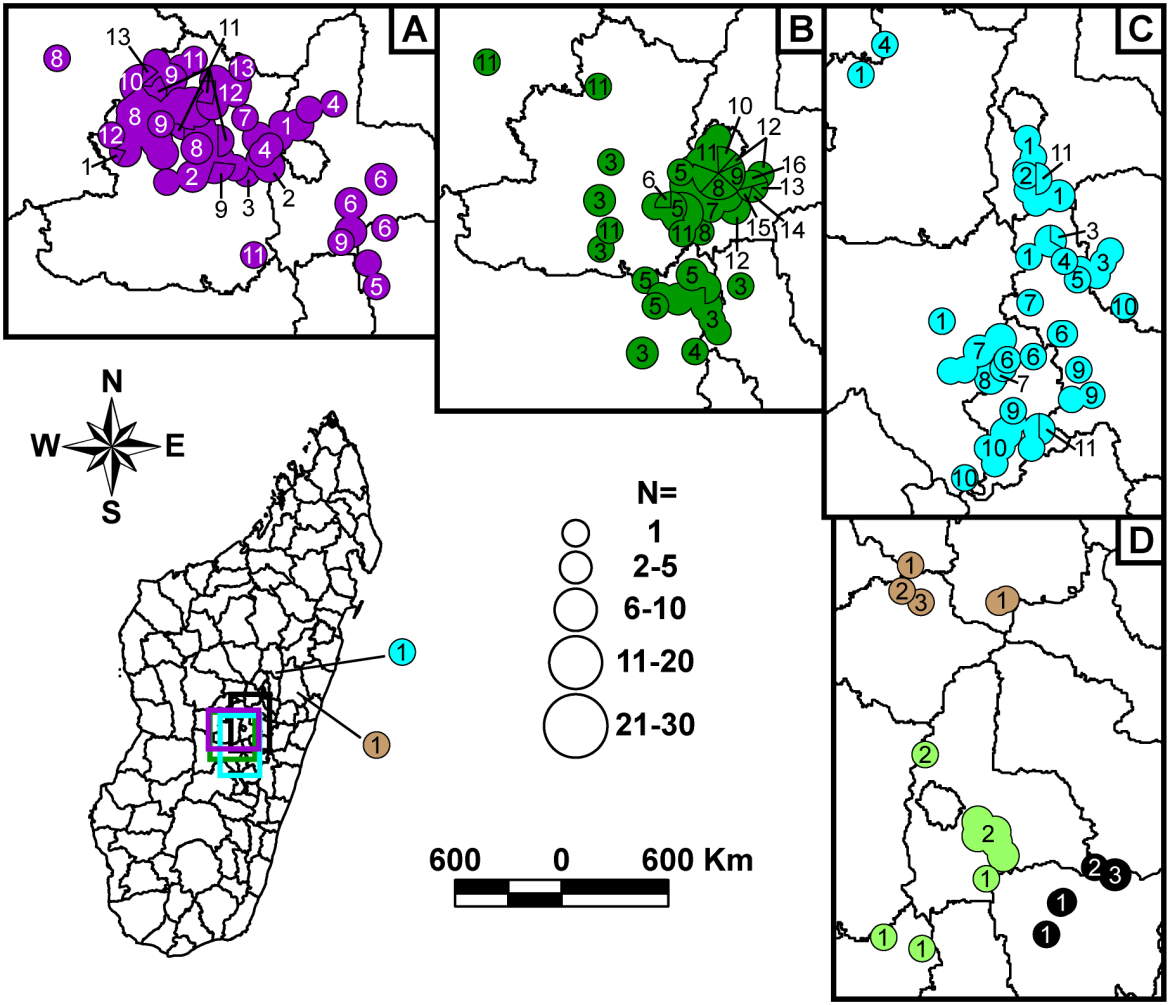
Geographic distributions of identified SNP nodes within Group II. Four map panels showing the geographic distributions of identified SNP nodes within Group II phylogenetic subgroups v (A), h (B), t (C), and u, w, and x (D) are presented, with symbols and colors as in Fig 5. A full map of Madagascar indicates the portion of Madagascar included in each of the expanded map panels, with each expanded section indicated by a colored rectangle corresponding to the color associated with one of the phylogenetic subgroups depicted in the corresponding expanded panel.

A SNP phylogeny was generated for all 773 samples using data from 42 informative SNPs from the 63 screened previously identified SNPs and 170 additional informative SNPs identified here from among the 188 potential new SNP targets (Fig 1A, S2 and S3 Tables). Neighbor-joining dendrograms based upon MLVA data were then constructed using MEGA6 [35] for each node containing >1 sample to further discriminate among samples. Subgroups were identified primarily based on SNPs, but also using MLVA for unresolved samples belonging to the basal k and d nodes (Fig 1). The geographic distributions of all of the identified subgroups were then mapped through time to determine temporal phylogeographic patterns using ArcGIS 10.2.1 for Desktop (ESRI, Redlands, CA) and geographic point data obtained from GeoPostcodes (http://www.geopostcodes.com/) for the fokontany and communes represented in the dataset (Figs 2, 3 and 4). Additional maps illustrating the geographic distributions of all of the identified SNP determined nodes were also generated (Figs 5 and 6).

### Estimation of divergence times

To determine if evolution of *Y*. *pestis* in Madagascar is operating under a molecular clock, we reconstructed a neighbor-joining phylogeny based upon the SNPs identified among the 31 strains sequenced here (S1 Table) and the previously published genomes for strains IP275 [GenBank: AAOS00000000] and CO92 [24]. We then uploaded the newick file, with associated dates of isolation, into TempEst [36], enforcing the selection of the best fitting root (CO92) and the correlation function. The correlation coefficient and R^2^ values were calculated, and we used a permutation test (10,000 permutations of distances) in R to determine if the observed correlation coefficient was better than would be expected by chance.

To estimate divergence times for subgroups of *Y*. *pestis* in Madagascar, we employed a Bayesian molecular clock method as implemented in the BEAST v1.8.0 software package [37]. Model selection analyses were carried out in MEGA 7.0.9 for the 33 included genomes, where the corrected Akaikes’s Information Criterion [38, 39] results were used to determine the best fitting models. The GTR model was found to be best fitting for the dataset. Because only variable sites were included in this analysis, we corrected for the invariant sites by specifying a Constant Patterns model in the Patterns List of the BEAST xml file (A’s: 1,219,459, C’s: 1,102,556, T’s: 1,217,289, and G’s: 1,114,076), and then also performed an uncorrected analysis for comparison. To determine the best fitting molecular clock and demographic model combinations for this dataset, path sampling [40] and stepping stone [41] sampling marginal likelihood estimators were employed [42, 43]. Model comparison analyses indicated that the combination of the uncorrelated lognormal molecular clock (UCLN) [44] and the Bayesian Skyride [45] models best fit the SNP dataset; however, timing estimates for both the TMRCA-All and TMRCA-Madagascar/Group I were starkly bimodal. We instead selected the more conservative UCLN-Constant model, which performed slightly worse in the model comparison, but incorporated fewer parameters. In addition, and without the incorporation of interior calibrations, usage of the UCLN-Constant model resulted in estimates of both the TMRCA-All and TMRCA-Madagascar/Group I clades that were historically supported (Table 1).

**Table 1:** Divergence time estimates for nodes of interest.

	Uncorrected			AB-Corrected		
	Mean	Median	95% CI	Mean	Median	95% CI
TMRCA-All (CO92 & Madagascar)	1857	1905	1722–1973	1880	1913	1754–1974
TMRCA-Madagascar/Group I	1928	1954	1855–1987	1927	1954	1864–1987
TMRCA-Group II	1949	1960	1889–1994	1951	1965	1889–1991

The SNP Ascertainment Bias Correction (AB-Corrected) data set has been corrected for the appropriate counts of invariant A’s, T’s, G’s, and C’s, allowing for the estimation of distances based on the true distribution of nucleotides for this data set. TMRCA: the most recent common ancestor.

For each dataset, four independent Markov chain Monte Carlo (MCMC) chains were run for 100 million generations each, with parameters and trees drawn from the posterior every 10,000^th^ step. Visual trace inspection and calculation of effective sample sizes was conducted using Tracer [46], confirming MCMC mixing within and among each of four replicate chains. LogCombiner [37] was used to merge the samples from each chain. The first 10% of each chain was discarded as burn-in, and then each chain was resampled every 40,000^th^ step. FigTree [47] was used to visualize the resulting phylogenies.

## Results

### Phylogenetic diversity of *Y*. *pestis* in Madagascar

There is considerable phylogenetic diversity among *Y*. *pestis* strains from Madagascar. The 170 new informative SNPs identified here (S2 Table) considerably expanded on previously published SNP phylogenies of *Y*. *pestis* in Madagascar [4, 18, 19]. The previously identified Groups I and II were still readily apparent, but with several additional lineages within these groups. Group I included the basal k node and seven lineages, five of which (j, l, q, r, and s) were previously described [18, 19] and two of which (y and z) were identified here (Fig 1A). Group II included the basal d node and six lineages, one of which (h) was previously described [19] and five of which (t–x) were identified here (Fig 1A). In addition to the seven novel lineages, additional resolution within the previously described h, j, q, and s lineages [18, 19] was also identified (Fig 1A). In all, 100 individual nodes were identified, providing considerable SNP resolution among the 773 Malagasy *Y*. *pestis* samples (Fig 1A, Table 2). MLVA provided additional resolution within the SNP determined nodes, with a resolving power range of 41%– 100% (average of 82%) for nodes with >1 sample (Table 2). Within the basal d and k nodes, MLVA identified one (II.D.1) and four (I.C, I.E, I.G, and I.L) additional phylogenetic subgroups, respectively (Fig 1B). These subgroups mostly corresponded to previously identified MLVA subgroups [17, 19] from which no strains have yet been sequenced, preventing further lineage identification using SNPs. In all, we identified 18 major subgroups among the 773 analyzed samples, including 13 SNP lineages (hereafter referred to as subgroups h, j, l, and q–z) and 5 MLVA subgroups (hereafter referred to as subgroups I.C, I.E, I.G, I.L, and II.D.1) (Fig 1).

**Table 2:** MLVA diversity within SNP nodes.

SNP Node	No. Samples	No. MLVA Genotypes	MLVA Resolving Power
d	10	10	100%
h03	17	14	82%
h04	1	1	100%
h05	17	11	65%
h06	1	1	100%
h07	25	15	60%
h08	6	4	67%
h09	3	2	67%
h10	2	1	50%
h11	18	8	44%
h12	5	5	100%
h13	1	1	100%
h14	1	1	100%
h15	1	1	100%
h16	3	2	67%
j01	9	8	89%
j02	14	12	86%
j03	36	30	83%
j04	1	1	100%
j05	8	6	75%
j06	1	1	100%
j07	16	8	50%
j08	4	3	75%
j09	5	4	80%
j10	2	2	100%
k	38	30	79%
l01	4	4	100%
l02	1	1	100%
q04	9	9	100%
q05	3	2	67%
q06	30	19	63%
q08	7	7	100%
q09	1	1	100%
q10	34	25	74%
q11	1	1	100%
q12	12	12	100%
q13	1	1	100%
q14	1	1	100%
r	5	4	80%
s03	23	21	91%
s04	6	6	100%
s05	34	15	44%
s08	34	14	41%
s09	24	11	46%
s10	10	9	90%
s11	2	2	100%
s12	1	1	100%
s13	11	10	91%
s14	10	10	100%
s15	4	3	75%
s16	2	1	50%
s17	1	1	100%
s18	4	4	100%
s19	9	7	78%
s20	6	5	83%
s21	3	3	100%
s22	1	1	100%
t01	17	15	88%
t02	1	1	100%
t03	5	5	100%
t04	2	2	100%
t05	1	1	100%
t06	4	4	100%
t07	8	8	100%
t08	2	2	100%
t09	4	4	100%
t10	11	9	82%
t11	2	2	100%
u01	3	3	100%
u02	1	1	100%
u03	2	2	100%
v01	7	4	57%
v02	17	15	88%
v03	1	1	100%
v04	4	4	100%
v05	2	2	100%
v06	7	6	86%
v07	1	1	100%
v08	39	36	92%
v09	13	10	77%
v10	3	2	67%
v11	10	7	70%
v12	10	9	90%
v13	2	1	50%
w01	3	3	100%
w02	16	10	63%
x01	4	4	100%
x02	1	1	100%
x03	1	1	100%
y01	1	1	100%
y02	1	1	100%
y03	6	5	83%
y04	2	2	100%
y05	2	2	100%
z01	1	1	100%
z02	1	1	100%
z03	5	5	100%
z04	3	2	67%
z05	1	1	100%
z06	2	1	50%

The number of samples within each SNP defined node in Fig 1A is indicated, along with the number of MLVA genotypes among those samples and the calculated resolving power of MLVA among the samples in each node.

As previously observed [18, 19], there was considerable congruence between the SNP and MLVA analyses. First, the previously identified congruence between lineages h, j, l, q, r, and s and the previously described MLVA subgroups II.B, I.J, I.H, I.B, I.F, and I.A, respectively, was still apparent [18, 19] (S2 Fig). Second, novel lineages t, v, and y corresponded with the previously identified MLVA subgroups II.A, II.C, and I.D, respectively [19] (S2 Fig). Third, novel lineages u, x, and z were first tentatively identified as new MLVA subgroups in the initial MLVA analysis (S2 Fig) used to select sequencing candidates and then confirmed using the new genome sequences and SNP analyses. Finally, the new lineage w corresponds to a subdivision within the previously identified MLVA subgroup II.D [19], with the other samples belonging to this MLVA subgroup remaining in the basal d node within identified MLVA subgroup II.D.1 (Fig 1, S1 Table, S2 Fig).

#### Geographic distribution

Overall, the geographic distributions of the 18 major subgroups were consistent with previous observations [17, 19] but with greater phylogeographic resolution thanks to the larger number of samples and use of finer scale fokontany geographic point data. Subgroup s remained the most geographically widespread, occurring in 23 of the 32 districts sampled here (Figs 2, 3, 4 and 5D, S1 Table). It continued to be concentrated in the northern central highlands in and around the capital of Antananarivo, but was also found further south. In addition, as previously determined [18, 19], it was also the subgroup responsible for the re-emergence of plague in the port city of Mahajanga during the 1990s (Fig 2). Individual SNP defined nodes within subgroup s displayed some additional spatial clustering, with newly identified nodes s10 –s11 found mostly in the south in districts Ambositra and southern Fandriana, s14 found mostly in districts Soavinandriana and Faratsiho, s15 –s16 isolated to district Faratsiho, and s17 –s18 and s20 –s22 found mostly in Antananarivo and the immediately adjacent district Manjakandriana. The geographic distributions of previously identified nodes s03 –s09 were consistent with previous observations [17, 18], with nodes s03 and s05 dispersed across the overall distribution of subgroup s, s04 found mostly in district Miarinarivo, and s08 –s09 found almost entirely in Mahajanga. Newly identified nodes s13 and s19 were also dispersed throughout the subgroup s distribution (Fig 5D). Overall, spatial clustering of the individual nodes was consistent with the SNP phylogeny, with phylogenetically close nodes clustered near to each other spatially (Figs 1A and 5D). Concordant with this observation, those nodes that tended to be more geographically dispersed were also generally more basal within the phylogeny and/or contained larger numbers of samples with substantial MLVA diversity (Fig 1A, Table 2); this suggests that additional whole genome sequencing of strains within these nodes would lead to further SNP discovery, node identification, and likely spatial clustering among the newly discovered nodes. Subgroup s was also temporally persistent, with at least one sample identified in almost every year of this 18 year analysis (Figs 2, 3 and 4, S1 Table). As such, this subgroup remained one of the most successful subgroups identified in Madagascar, persisting over many years over a large geographic area and in several different species involved in plague transmission (Figs 2, 3 and 4, S1 Table).

Other Group I subgroups also showed persistence through time, despite smaller sample sizes. Few of the novel samples were found to belong to subgroups l, r, y, I.C, I.E, I.G, and I.L, likely due to the lack of sampling emphasis in the geographic areas where these subgroups were found (S1 Table). However, temporal analysis of these subgroups indicated persistence of most of these subgroups in the same geographic locations for periods of time ranging from 4 to 13 years, with 0 to 3 samples identified in a given year for individual subgroups (Figs 2, 3 and 4, S1 Table). Similar to subgroup s, the individual nodes identified within subgroups l and y also exhibited some additional spatial clustering that was consistent with the SNP phylogeny (Figs 1A and 5B). Only two subgroups were restricted to a single sampling year: subgroup I.C with two samples identified in district Bealanana in 2000 and subgroup I.L with 17 samples identified in district Ambalavao in 2007 (Figs 2 and 3, S1 Table). However, neither of these geographic areas were particularly well sampled, which may have contributed to the failure to observe these subgroups in other sampling years.

Consistent with previously identified subgroups, the three entirely new subgroups identified here were also found to occupy distinct geographic locations and to persist through time in those areas. Subgroup z, the one entirely new subgroup identified within Group I (Fig 1A), was concentrated in district Antanifotsy and neighboring Antsirabe II, with representatives identified in this area over a 12 year period (Figs 2, 3, 4 and 5B, S1 Table). Two entirely new subgroups, u and x, were identified within Group II (Fig 1A). Subgroup u was restricted to the northern half of district Fandriana, with representatives identified in this area over a 14 year period. Subgroup x samples were also identified over a 14 year period and were mostly found in the area defined by northern Faratsiho and southern Miarinarivo and Arivonimamo (Figs 2, 3, 4 and 6D, S1 Table). Individual nodes within these three new subgroups displayed some additional spatial clustering, consistent with the SNP phylogeny (Figs 1A, 5B and 6D), and similar to what was observed for subgroups l, s, and y. Indeed, these new subgroups may be the primary phylogenetic types found in their respective geographic areas, which were not well sampled in previous analyses [17, 19]. However, this requires confirmation, as sampling in these areas was still limited in this analysis (Figs 2, 3 and 4, S1 Table), and there was at least some geographic overlap between the newly identified subgroup x and the highly successful subgroup s (Figs 2, 3 and 4).

### Betafo region

A far more robust phylogeographic analysis was possible in the heavily sampled Betafo region (districts indicated with yellow shading and parts of districts indicated with yellow striped shading in S1 Fig). Several geographically distinct subgroups were previously identified in this region [17, 19]. These subgroups, identified here as subgroups h, j, t, v, w, and II.D.1, were also observed in this analysis, and showed persistence in the same geographic areas over most of the 18 year study period (Figs 2, 3, 4, 5A and 6). Specific geographic distributions of the individual subgroups were consistent with previous observations [17, 19] but much expanded. In summary, subgroup j dominated in district Mandoto with some overlap with subgroup v, which dominated in the neighboring Betafo district (Figs 2, 3, 4, 5A and 6A). Subgroup h was most prominent southeast of subgroup v, being found mostly in the southwestern part of district Antsirabe II, but also in Antsirabe I, southeastern Betafo, and northeastern Ambatofinandrahana (Figs 2, 3, 4 and 6B). Subgroup w was also found in district Antsirabe II and northeastern Ambatofinandrahana, but, in general, was further east than subgroup h (Figs 2, 3, 4 and 6D). Subgroup t was mostly found further south, in districts Manandriana, Ambositra, Fandriana, and eastern Ambatofinandrahana, but also occurred in the southern part of district Antsirabe II, mostly in between subgroups h and w (Figs 2, 3, 4 and 6C). Subgroup II.D.1 was on the periphery of the Betafo region in eastern Ambatofinandrahana, and was, in general, further south than subgroup h and further west than subgroup t (Figs 2, 3 and 4).

Geographic patterns among the individual SNP defined nodes within subgroups h, j, t, and v were less distinct. Similar to other subgroups, the overall geographic distributions of the individual nodes within each of these subgroups were consistent with the SNP phylogeny, with phylogenetically close nodes clustered near to each other spatially (Figs 1A, 5A, 6A, 6B and 6C). However, compared to other subgroups, there was far more overlap and fewer distinct geographic patterns among the individual nodes, particularly for subgroups h, j, and v. All three of these subgroups contained several nodes that were dispersed across the overall geographic distribution of their respective subgroups, including h03, h05, and h11 for subgroup h, j01 –j03 and j05 for subgroup j, and v09 and v11 –v13 for subgroup v (Figs 5A, 6A and 6B). Similar to the geographically dispersed nodes within subgroup s, most of these nodes were more basal within the phylogeny and/or contained larger numbers of samples and considerable MLVA diversity (Fig 1A, Table 2). The other nodes within these subgroups were more spatially restricted, but were also less geographically distinct compared to the spatially restricted nodes in other subgroups (Figs 5 and 6). The individual nodes identified within subgroup t were more geographically distinct, with t02 located in southwestern Antsirabe II, t03 –t05 found mostly in northern Ambositra and western Fandriana, t06 –t08 found further south in eastern Ambatofinandrahana, northern Manandriana, and western Ambositra, and t09 –t11 found predominantly even further south in southeastern Ambatofinandrahana, southern Manandriana, and southwestern Ambositra. Basal node t01 was more geographically dispersed, but was concentrated in the northernmost part of the geographic range of subgroup t in southwestern Antsirabe II, along with t02 (Fig 6C).

### Moramanga region

Samples from the Moramanga region (districts indicated with purple shading and parts of districts indicated with purple striped shading in S1 Fig), the other heavily sampled region in this analysis, showed much less phylogenetic diversity than samples from the Betafo region. As previously observed [17, 19], the Moramanga region was dominated by subgroup q, with very few samples from this region belonging to any other subgroup (Figs 2, 3 and 4). In contrast to previous analyses, this analysis also revealed substantial phylogeographic structure among the individual SNP determined nodes within this subgroup, with q06 found predominantly in district Manjakandriana, the southern tip of Anjozorobe, and a small area in western Moramanga, q05 and q08 –q09 found mostly further north in district Anjozorobe, q10 –q11 found predominately in eastern Moramanga, a single q14 sample found in southern Moramanga, and q12 found in the northernmost part of the geographic distribution of subgroup q, in districts Andilamena, Tsaratanana, and northeastern Anjozorobe. Node q04, a more basal node in the q subgroup, was the least geographically defined, with representatives identified in between the distributions of q06 and q10 in district Moramanga, and also in a small area in eastern Anjozorobe in the midst of some q05, q08, and q12 representatives (Fig 5C). Previous analyses were consistent with these phylogeographic patterns, but far less defined due to the much more limited sample sizes and lower phylogenetic resolution in those analyses [17, 19]. Similar to the other subgroups, subgroup q also displayed temporal persistence within its geographic range in the northeastern central highlands over the 18 year study period (Figs 2, 3 and 4).

### Temporal variation

**Fig 7:**
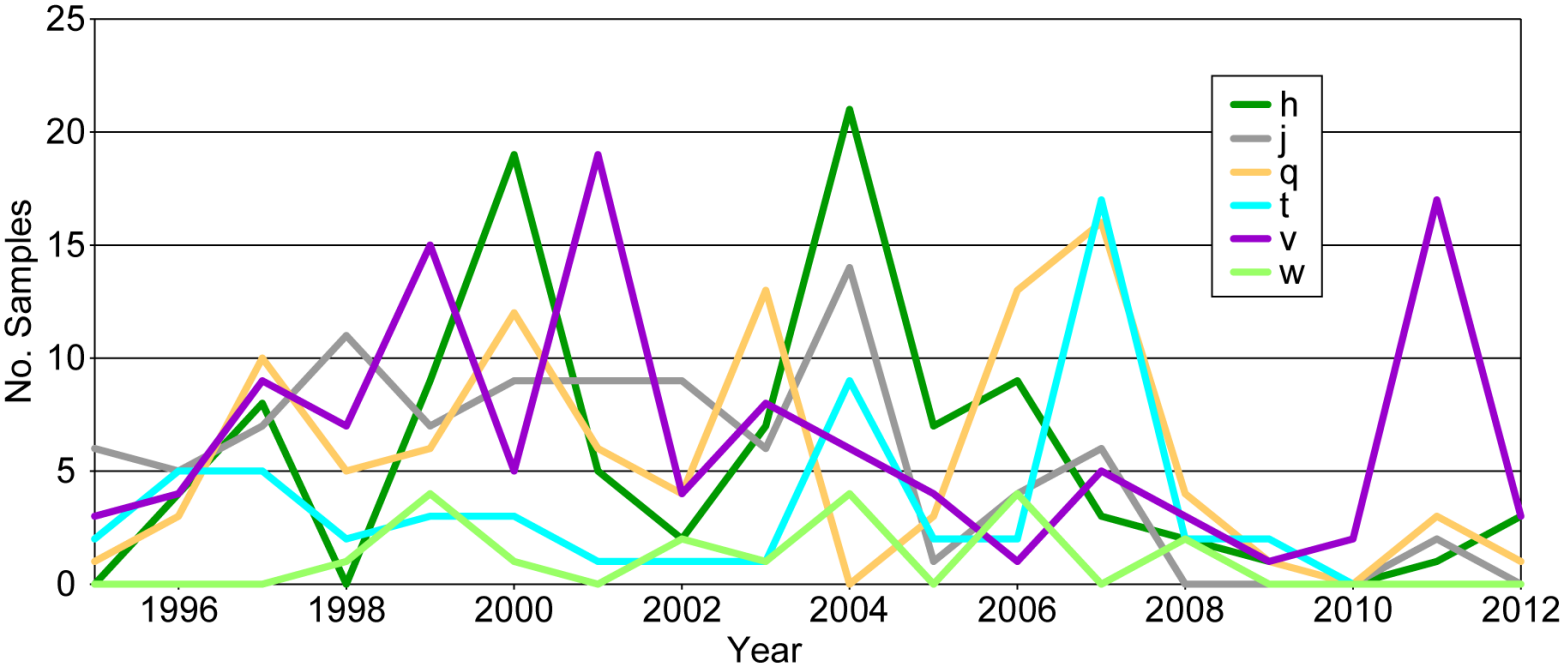
Variation in sampling frequency over time. The number of samples identified each year in this analysis for each *Yersinia pestis* subgroup found predominantly in the well-sampled Betafo and Moramanga regions.

Although there was an overall pattern of temporal persistence in the same geographic areas over the 18 year study period for most of the identified subgroups, many subgroups varied in the frequency of samples identified from year to year (Figs 2, 3 and 4, S1 Table). Although the extent of this variation could not be determined for many of the subgroups due to uneven sampling in some geographic areas, the comprehensive sampling of the Betafo and Moramanga regions allowed for a closer examination of this variation for the subgroups found predominantly in these regions. These subgroups (h, j, q, t, v, and w) varied in the number of samples identified per year for each subgroup, with high and low sampling years observed for each subgroup. Moreover, this variation did not follow the same pattern among all of the subgroups. For example, the number of subgroup v samples identified in 1999 and 2001 were relatively high compared to the intervening year, in 2000. In contrast, subgroups h and q experienced peaks in sample identification in 2000 and lower frequencies of sample identification in the bracketing years of 1999 and 2001 (Fig 7).

### Dispersal events

In contrast to the overall pattern of geographic and temporal stability of the 18 identified subgroups, we observed some subgroup representatives in geographic areas outside their primary geographic range. Most interesting were several examples of more long distance dispersal events, such as five samples of subgroup q (found primarily in the northeastern central highlands, Fig 5C) that were isolated in and around the Betafo region in 1996 (N = 1), 1997 (N = 2), and 2006 (N = 2), respectively (Figs 2, 3, 4 and 5C, S1 Table). Interestingly, these five samples included representatives of five different SNP defined nodes within this subgroup (Fig 5C, S1 Table). All of the other samples assigned to these five nodes were isolated in the northeastern central highlands, suggesting that these nodes evolved there. Together, these patterns strongly suggest that the five samples of subgroup q isolated in the Betafo region were the result of independent dispersal events from the typical geographic range of subgroup q in the northeastern central highlands. Indeed, many of the occurrences of subgroups outside of their typical geographic ranges were likely due to independent dispersal events rather than an initial dispersal event followed by localized establishment of the transferred subgroup, as there was little to no evidence of persistence of a “transferred” subgroup in a non-typical geographic area. However, it should also be noted that this lack of evidence could have been due to inadequate sampling that failed to detect any low level persistence of these “transferred” subgroups. Regardless, any dispersal events, particularly over long distances, are likely human-mediated and either related to the accidental transport of rats and fleas along with legitimate shipments, or could also be related to humans who were infected in a location distant from where they sought medical attention. Indeed, the black rat has been shown to have a range of only 40–50 m during normal activities, with travel up to only ~350 m in pursuit of resources [48], and so rat dispersal alone is unlikely to account for these types of observed transfers of *Y*. *pestis* genotypes.

Other examples of potential dispersal events involved shorter distances and could have multiple causes. These included samples of subgroup j occasionally being isolated within the typical geographic range of neighboring subgroup v and vice versa, as well as other similar crossover type events that occurred among the other densely packed and phylogenetically diverse subgroups found in the highly active Betafo region (Figs 2, 3 and 4). These examples may represent dispersal events over shorter distances that could have been rat- or human-mediated. Alternatively, these examples might not reflect dispersal events at all but could, instead, indicate subgroups that are actually established in more than one geographic area but are very rare in the area in which the “dispersal event” appears to have occurred, at least as represented by human derived samples.

### Divergence times

To determine whether or not *Y*. *pestis* in Madagascar is evolving according to a molecular clock, we performed a linear regression and found that 28% (R^2^ = 0.28) of the variation in root-to-tip distances could be explained by time (S3B Fig). Permuting the distances 10,000 times revealed that the observed correlation coefficient, R = 0.527, was better than 99% of all randomly generated correlation coefficients (S3C Fig). These analyses revealed that although strict molecular clock methods were not appropriate due to the limited, albeit statistically significant, variation of distance explained by time, relaxed molecular clocks were well-suited for divergence time estimation.

**Fig 8:**
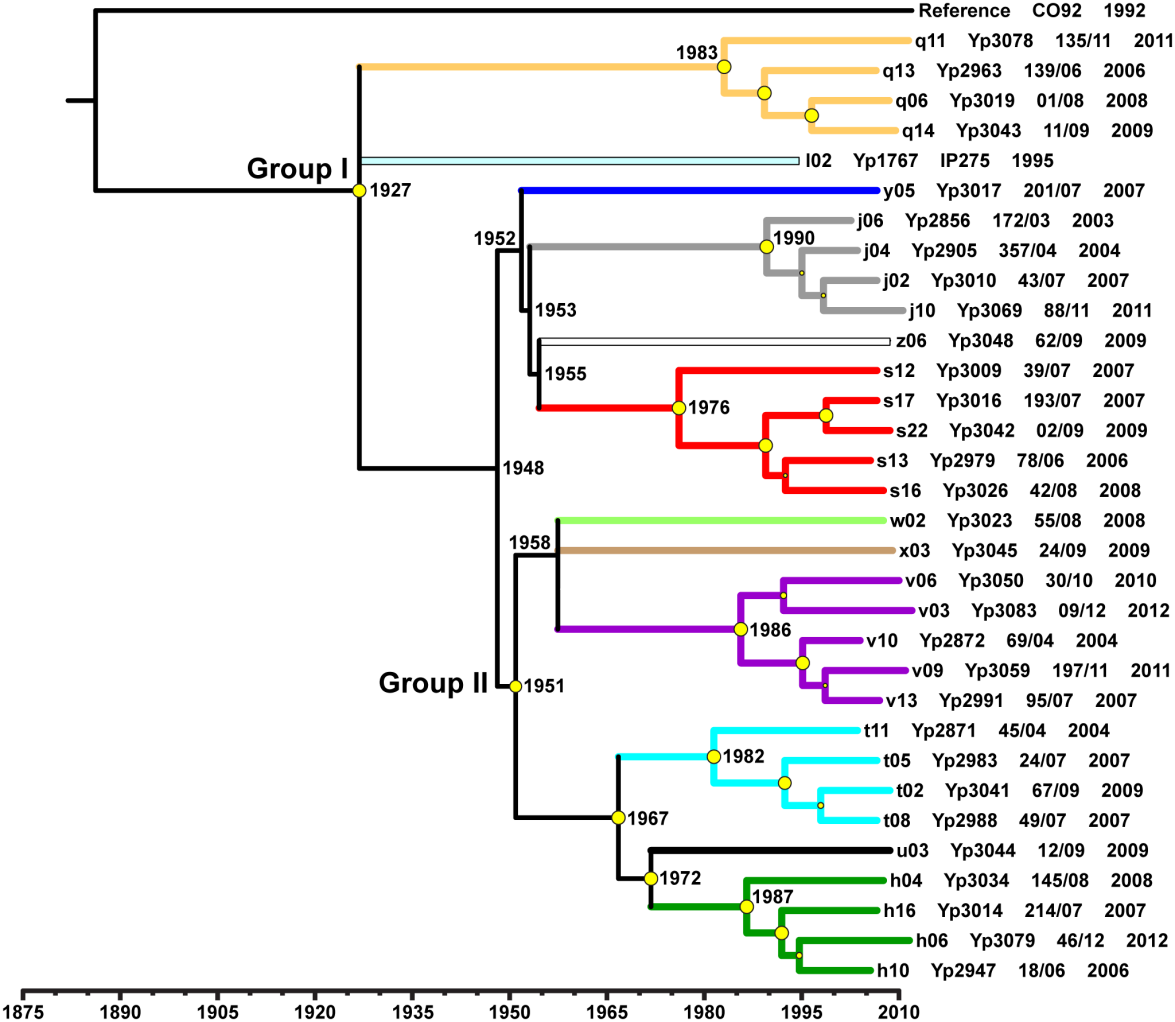
Maximum clade credibility phylogeny, reconstructed in BEAST, with mean divergence times for whole genome sequences from 32 Malagasy *Yersinia pestis* strains and reference strain CO92. A maximum clade credibility phylogeny based upon SNPs identified among the 31 Malagasy *Y*. *pestis* strains sequenced here and the previously sequenced Malagasy strain IP275 and reference strain CO92. Malagasy strain branches are labeled with the SNP nodes from Fig 1, the strain IDs from S1 Table, and the year of isolation of the strain. Colors of the clades and/or branches indicate identified subgroups and correspond to the subgroup color designations in Fig 1. A timeline indicates the estimated mean divergence times for the various branch points. Estimated mean divergence times for various nodes of interest are also indicated on the phylogeny. Yellow circles indicate the posterior probabilities for each of the clades, where larger circles indicate higher confidence.

Bayesian estimation of divergence times using the ascertainment bias correction with a relaxed molecular clock and constant population size model, revealed that CO92 and the Malagasy strains diverged from their MRCA in 1880 (mean date; Table 1, Fig 8), which was consistent with a split following the onset of the third pandemic in 1855 in Yünnan, China [2], and prior to the introduction of *Y*. *pestis* to Madagascar in 1898 [12]. The estimated mean TMRCA for the Malagasy strains, and therefore for the basal Group I, was 1927 (Table 1, Fig 8), just 29 years after the introduction of plague to Madagascar. Importantly, the confidence intervals for this TMRCA (95% CI: 1864–1987, Table 1) did not stretch back in time beyond the third pandemic, indicating that our divergence time analysis, using only tip calibrations, was supported by historical events. The mean divergence of the Group II Malagasy strains from Group I was estimated to have occurred in 1951 (Table 1, Fig 8). Indeed, the estimated mean divergence times for most of the subgroups identified in this analysis were after 1950, which was when several successful plague control methods were implemented in Madagascar that led to a large decrease in the numbers of human cases [9]. Likewise, the steady increase in human plague cases that began in the 1980s in Madagascar [9] was consistent with the estimated mean divergence times for the different lineages within those subgroups with multiple whole genome sequence representatives, which ranged from 1976 to 1990 (Fig 8). A final observation from this analysis was that although the mean and median divergence times for the deeper CO92 and Malagasy strains were impacted by the SNP ascertainment bias correction, the confidence intervals for all divergence time estimates, and also the mean and median divergence times for the more recent Group I and Group II divergences, were nearly identical (Table 1).

## Discussion

Plague continues to be a significant public health concern in Madagascar, with hundreds of human cases reported annually [9, 49]. Human cases exhibit strong seasonality as well as spatial and temporal variation in the affected fokontany. Most human cases occur from October to April during the warm rainy season [8, 50, 51] and different fokontany are affected in different years, with some fokontany unaffected despite nearby fokontany having cases [48]. The seasonality of plague is linked to population dynamics of the black rat and its flea vectors, with onset of the plague season in October coinciding with the minimum abundance of rats and maximum abundance of fleas [8, 10, 11]. The basis for the spatial and temporal variation in affected fokontany is less clear [48], but could be related to similar ecological factors or stochastic forces. Our temporal phylogeographic analysis of 773 *Y*. *pestis* samples from 32 districts in Madagascar, collected over 18 years, with an emphasis on the Betafo and Moramanga regions, provides insight into this and other aspects of plague ecology in Madagascar.

Previous analyses have suggested that *Y*. *pestis* in Madagascar is maintained in multiple, geographically and phylogenetically distinct subpopulations likely sustained by the black rat [17–19]. Our analysis is consistent with this hypothesis and suggests that these subpopulations are spatially and temporally stable, with the same phylogenetic types persisting in the same geographic locations over a period of almost two decades (Figs 2, 3 and 4). This observed temporal phylogeographic pattern suggests that persistent endemic cycles of *Y*. *pestis* transmission within local areas are responsible for the long term maintenance of plague in Madagascar, rather than repeated episodes of wide scale epidemic spread. Indeed, there is little evidence for frequent, widespread selective sweeps of individual genotypes. Dispersal events do occur, but seldom appear to result in the successful establishment of a new genotype in a new location (Figs 2, 3 and 4).

The failure of a dispersal event to result in successful ecological establishment may be strongly affected by the presence or absence of an existing locally established and cycling genotype. For example, Mahajanga was likely free of *Y*. *pestis* when a long distance dispersal event from the central highlands allowed for at least the temporary successful establishment of subgroup s in this city in the early 1990s [18]. Another, related factor that likely assists in plague establishment is the presence of a high abundance of susceptible hosts [5, 52]. This very likely played a role in the Mahajanga outbreaks, which began in an area with poor hygiene and large numbers of rats and shrews [13, 14, 53]. Similarly to Mahajanga, the apparent spread of subgroup s from its presumed origin in district Ambositra to Antananarivo and the surrounding areas [18, 19] may have been facilitated by a lack of locally circulating genotypes and perhaps an abundance of susceptible hosts. Supporting this idea is the fact that for ~30 years following the successful plague control campaigns of the 1950s there were only 20 to 50 human plague cases reported per year in Madagascar, and in Antananarivo specifically no cases were reported between 1953 and 1978 [9]. Following this, the number of annual human plague cases increased steadily [9], with notable subgroup s-linked outbreaks in Antananarivo and Mahajanga in the 1990s [9, 18, 19]. This suggests that there may have been an open niche in Antananarivo and the surrounding areas that subgroup s was able to occupy following a fortuitous dispersal event, similar to what happened in Mahajanga. Interestingly, the estimated mean divergence time for the various lineages within subgroup s was 1976 (Fig 8), which is consistent with this timeline. Intriguingly, the rodent population in Antananarivo at this time consisted of approximately 80% *Rattus norvegicus*, a host not usually thought of as highly susceptible to plague [53]. By the late 1990s, *R*. *norvegicus* made up 95% of the rodent population and both *R*. *rattus* and *R*. *norvegicus* from Antananarivo displayed high levels of plague resistance [54]. Thus, if subgroup s has only been present in Antananarivo since the late 1970s, it succeeded in becoming established and persisting in a relatively resistant host population. Previous studies have also suggested that subgroup s may possess some adaptive advantage affecting its ability to become established following a dispersal event [18, 19]. If so, this advantage does not appear to have enabled subgroup s to further expand its geographic range [17, 19] during the 18 year period of this analysis, despite likely dispersal events of this subgroup to other areas (Figs 2, 3, 4 and 5D). It is possible that the presence of other locally established and cycling genotypes (i.e., an occupied niche), as documented here, inhibited the establishment of this or other transferred genotypes during the 18 year period of this analysis (Figs 2, 3 and 4).

The Betafo and Moramanga regions emphasized in this analysis exhibited distinct differences in observed phylogenetic diversity that may be related to landscape differences between these two regions. The Moramanga region consists of a wide valley along the Mangoro River that contains large and fragmented forested areas and gradually decreases in elevation from north to south. The Betafo region, in contrast, consists of a much more diverse landscape. District Mandoto in the western portion of this region consists of a plateau area with rolling hills and a fairly flat relief. To the east, district Betafo is more rugged, with relatively large changes in elevation between fokontany. District Antsirabe is even more mountainous, with fokontany located in deep valleys separated by high ridges [55]. Interestingly, this landscape heterogeneity was mirrored in the observed phylogenetic diversity for these regions. The relatively homogenous and level landscape of Moramanga was dominated by a single subgroup, q (Figs 2, 3, 4 and 5C). Similarly, the relatively flat landscape of district Mandoto within the Betafo region was also dominated by a single subgroup, j (Figs 2, 3, 4 and 5A). In contrast, the more heterogeneous landscape of the rest of the Betafo region, characterized by much greater variation in elevation, contained much more phylogenetic diversity, with at least four distinct subgroups (h, t, v, and w) identified in close proximity (Figs 2, 3, 4 and 6). This suggests that landscape plays a role in maintaining the multiple geographically and phylogenetically distinct subpopulations of *Y*. *pestis* identified in Madagascar, likely by limiting the potential for dispersal of the black rat and its fleas. Indeed, population genetics studies of the black rat in Madagascar are consistent with this hypothesis, with rat populations from landscapes characterized by greater topographical relief showing greater genetic structure than rat populations from flatter areas [55].

There is strong evidence that *Y*. *pestis* population sizes vary through time. Population expansions and contractions related to the alternating epizootic and enzootic cycles that characterize *Y*. *pestis* are likely the basis of the highly variable molecular clock rate observed across the worldwide *Y*. *pestis* phylogeny [3]. In Madagascar, the high and low plague seasons are associated with similar *Y*. *pestis* population expansions and contractions, as indicated by higher levels of both *Y*. *pestis* seroprevalence in rats [48] and numbers of human cases [50, 51] during the high plague season compared to the low season. A phylotemporal analysis of the Mahajanga outbreaks of the 1990s provided additional evidence, revealing a striking pattern of diversity generation and loss during and after each seasonal plague outbreak, consistent with seasonal population expansions and inter-seasonal population contractions [18]. In addition to these seasonal variations in *Y*. *pestis* population size, our analysis suggests that there is also likely variation in the magnitude of a population expansion during a given epizootic cycle in Madagascar, and that this variation is not consistent among the different *Y*. *pestis* subpopulations maintained in Madagascar. Specifically, we observed variation in sampling frequency from year to year for the subgroups found predominantly within the well-sampled Betafo and Moramanga regions. Assuming that the identified samples were representative of the underlying populations of these subgroups, this suggests that the magnitude of a seasonal *Y*. *pestis* population expansion varies from year to year. We also observed that the high and low sampling years observed for each subgroup were not consistent among subgroups. Together, these observations suggest that local, underlying ecological factors may affect the magnitude of seasonal population expansions of individual subgroups from year to year and, consequently, whether or not a subgroup was sampled in a given year. Many such potential ecological factors have been identified in Madagascar. Variation in elevation and associated temperature fluctuations are strongly associated with shifts in human plague seasonality and are thought to affect development of the flea vector and the efficiency of flea blockage by *Y*. *pestis* [8, 51]. Rat reproductive and migration patterns are influenced by similar seasonal climatic changes and related resource availability, particularly with regards to agricultural crops [48]. Local changes in these or other factors likely affect the population dynamics of the various *Y*. *pestis* subgroups established in Madagascar, which, in turn, likely affect the likelihood of observing human cases in a particular fokontany during a particular year.

Persistent endemic cycles of *Y*. *pestis* transmission within local areas of Madagascar result in strong, consistent spatial structuring that persists through time. Landscape likely influences local diversity of *Y*. *pestis*, with increased topographical relief associated with increased levels of localized differentiation, and the maintenance of multiple phylogenetically distinct subpopulations even within relatively short geographic distances. Dispersal events rarely appear to result in the establishment of a transferred genotype in a new location, possibly due to the presence of an existing locally cycling and established genotype. Local ecological factors in the geographic ranges occupied by individual *Y*. *pestis* subpopulations likely affect the dynamics of individual subpopulations, and the associated likelihood of observing human plague cases in a given year in a particular fokontany. Altogether, the ecology and epidemiology of *Y*. *pestis* in Madagascar are highly dynamic, affected by a variety of factors.

## Supporting information

S1 Fig Map of Madagascar. The 32 districts (shaded and numbered 1–32) where *Yersinia pestis* samples analyzed in this study were collected are indicated. The core districts included in the heavily sampled Betafo and Moramanga regions are shaded yellow and purple, respectively. The neighboring districts where additional samples belonging to the subgroups dominating the core districts were identified are indicated by yellow and purple striped shading for the Betafo and Moramanga regions, respectively.
(TIF)

S2 Fig Sample selection for whole genome sequencing. MLVA phylogenies of 458 and 315 Group I (A) and Group II (B), respectively, Malagasy *Y*. *pestis* samples used to select candidates for whole genome sequencing. The MLVA phylogenies consist of neighbor-joining dendrograms constructed in MEGA6 [35] using mean character based distance matrices. Each MLVA clade corresponding to an identified subgroup is collapsed, colored according to Fig 1, and labeled. Labels include the SNP defined lineage or basal node, the corresponding MLVA subgroup, and the number of samples from that clade selected for whole genome sequencing. An “*” marks the MLVA clades that were tentatively identified as potential new subgroups and later confirmed through whole genome sequencing and SNP analysis.
(EPS)

S3 Fig Detection of a temporal signal. (A) Table showing each taxon name (tip), the date of isolation, and distance of each tip from the root (CO92). (B) Linear regression to determine how correlated root to tip distance is with date of isolation. (C) Random distribution of 10,000 permutations, where root to tip distances were shuffled each time, and corresponding correlation coefficients are plotted along the x-axis (blue). The observed correlation coefficient (sqrt of 0.278 from B) is plotted in yellow. The observed value is greater than 99% of all random values (i.e. better than expected by random chance alone).
(PDF)

S1 Table Samples in this study. (XLSX)

S2 Table SNPs analyzed in this study. (XLSX)

S3 Table Primers used in SNP amplicon sequencing. (XLSX)
