# Supplementary figures and images for: Temporal phylogeography of Yersinia pestis in Madagascar: Insights into the long-term maintenance of plague
Source: PLoS Negl Trop Dis. 2017 Sep 5;11(9):e0005887. doi: 10.1371/journal.pntd.0005887 (PMC5600411; doi:10.1371/journal.pntd.0005887)

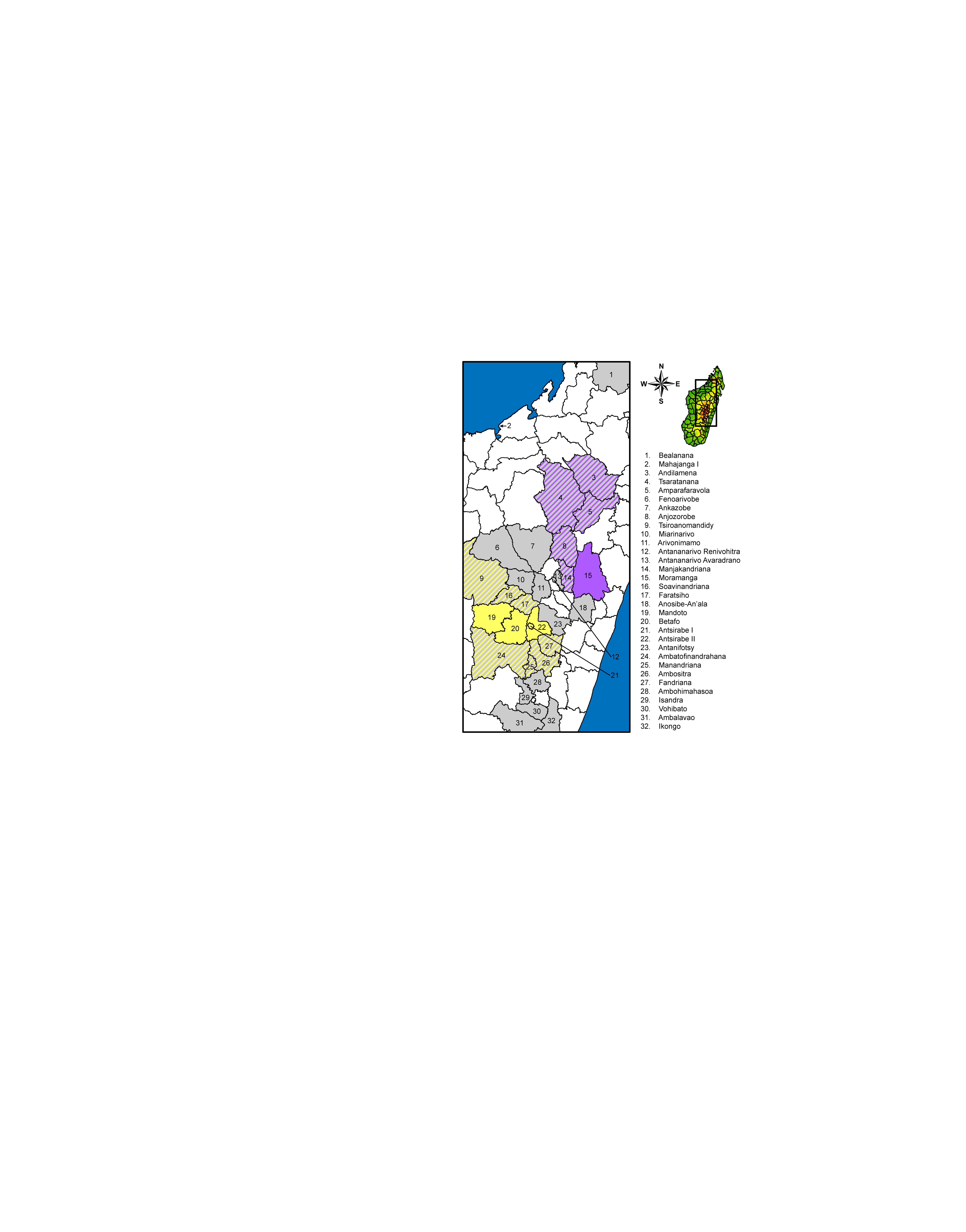

Supplement: S1 Fig — The 32 districts (shaded and numbered 1–32) where Yersinia pestis samples analyzed in this study were collected are indicated. The core districts included in the heavily sampled Betafo and Moramanga regions are shaded yellow and purple, respectively. The neighboring districts where additional samples belonging to the subgroups dominating the core districts were identified are indicated by yellow and purple striped shading for the Betafo and Moramanga regions, respectively. (TIF) [file pntd.0005887.s001.tif]
