# Supplementary material for: Temporal phylogeography of Yersinia pestis in Madagascar: Insights into the long-term maintenance of plague
Source: PLoS Negl Trop Dis. 2017 Sep 5;11(9):e0005887. doi: 10.1371/journal.pntd.0005887 (PMC5600411; doi:10.1371/journal.pntd.0005887)

A

Root to tip distances

tip	date	distance
CO92	1992	0
Yp3016	2007	0.187425
Yp3042	2009	0.207046
Yp3026	2008	0.2064
Yp2979	2006	0.180758
Yp3009	2007	0.193704
Yp3017	2007	0.198363
Yp2963	2006	0.215405
Yp3078	2011	0.225177
Yp3019	2008	0.213996
Yp3043	2009	0.215779
Yp3048	2009	0.194128
Yp2856	2003	0.194363
Yp2905	2004	0.191381
Yp3010	2007	0.188107
Yp3069	2011	0.202091
IP275	1995	0.22056
Yp3023	2008	0.194563
Yp3045	2009	0.194838
Yp3050	2010	0.181486
Yp3083	2012	0.188664
Yp2872	2004	0.180998
Yp2991	2007	0.191697
Yp3059	2011	0.185237
Yp2983	2007	0.184644
Yp3041	2009	0.181849
Yp2871	2004	0.206262
Yp3044	2009	0.182406
Yp3079	2012	0.183719
Yp3014	2007	0.199922
Yp2947	2006	0.195331
Yp3034	2008	0.216492
Yp2988	2007	0.127168

B

Correlation: Distance and Date

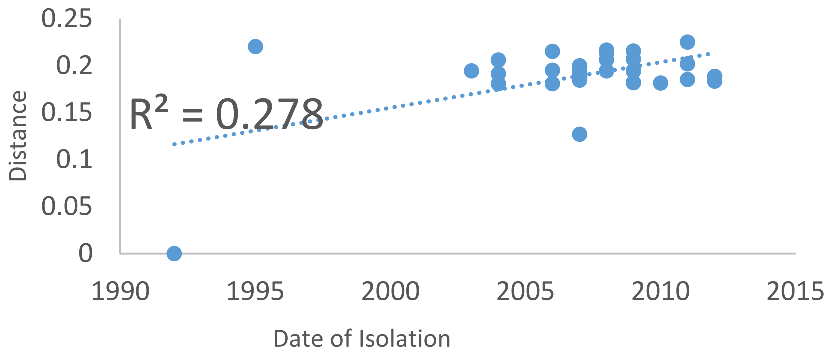

C

Density Distribution of Correlation Coefficients Calculated from Permuted Distances

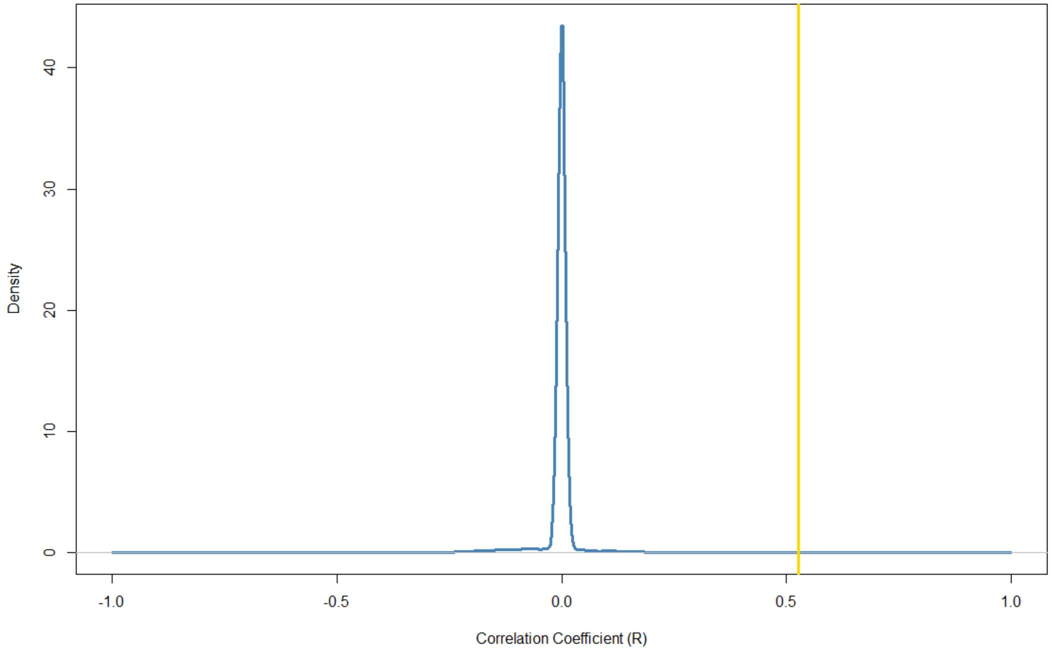

Supplement: S3 Fig — (A) Table showing each taxon name (tip), the date of isolation, and distance of each tip from the root (CO92). (B) Linear regression to determine how correlated root to tip distance is with date of isolation. (C) Random distribution of 10,000 permutations, where root to tip distances were shuffled each time, and corresponding correlation coefficients are plotted along the x-axis (blue). The observed correlation coefficient (sqrt of 0.278 from B) is plotted in yellow. The observed value is greater than 99% of all random values (i.e. better than expected by random chance alone). (PDF) [file pntd.0005887.s003.pdf]
